# National Evaluation of Emergency Medical Services Clinician Burnout and Workforce-Reducing Factors

**DOI:** 10.1016/j.acepjo.2024.100024

**Published:** 2025-01-10

**Authors:** Jonathan R. Powell, Christopher B. Gage, Remle P. Crowe, Laura J. Rush, Sarah R. MacEwan, Graham Dixon, Ann Scheck McAlearney, Ashish R. Panchal

**Affiliations:** 1 National Registry of Emergency Medical Technicians, Columbus, Ohio, USA; 2 Division of Epidemiology, College of Public Health, The Ohio State University, Columbus, Ohio, USA; 3 Department of Emergency Medicine, The Ohio State University Wexner Medical Center, Columbus, Ohio, USA; 4 ESO Solutions, Inc, Austin, Texas, USA; 5 The Center for the Advancement of Team Science, Analytics, and Systems Thinking in Health Services and Implementation Science Research (CATALYST), College of Medicine, The Ohio State University, Columbus, Ohio, USA; 6 Division of General Internal Medicine, College of Medicine, The Ohio State University, Columbus, Ohio, USA; 7 Department of Family and Community Medicine, College of Medicine, The Ohio State University, Columbus, Ohio, USA

**Keywords:** emergency medical services, burnout, workforce, public health, paramedic, emergency medical technician

## Abstract

**Objectives:**

Limited recent national data exist measuring burnout levels among emergency medical services (EMS) clinicians and the potential impact of burnout on workforce strength and stability. We aimed to measure current EMS burnout and its association with workforce-reducing factors.

**Methods:**

In April 2022, a random sample of nationally certified EMS clinicians was sent a survey that included the Copenhagen Burnout Inventory (CBI) to assess for burnout in 3 dimensions: personal, work-related, and patient-related. Descriptive statistics (count and percentage) and multivariable logistic regression (odds ratio [OR] and 95% CI) were used to identify and measure each domain of burnout and the relationship between these domains and workforce-reducing factors.

**Results:**

From 1838 survey responses (9% response rate), prevalence of EMS burnout was high in the personal (52%), work-related (49%), and patient-related (23%) domains. Burnout was higher for paramedics than for emergency medical technicians across all domains. Higher odds of reporting 10 or more sick days was observed for those with personal (OR, 2.66; 95% CI, 1.70-4.15), work-related (OR, 1.99; 95% CI, 1.31-3.01), or patient-related burnout (OR, 1.85; 95% CI, 1.20-2.86). Higher odds of reporting likelihood to leave the EMS profession was observed for those with personal (OR, 3.06; 95% CI, 2.16-4.33), work-related (OR, 3.34; 95% CI, 2.35-4.74), or patient-related burnout (OR, 3.42; 95% CI, 2.39-4.90).

**Conclusion:**

Nationally certified EMS clinicians demonstrated high burnout in 2022. Combined with increased absenteeism and intent to leave the profession associated with these high levels of burnout, these findings suggest that a renewed and deliberate focus on EMS clinician well-being is needed to ensure job satisfaction and workforce stability.

**The Bottom Line**

Emergency medical services clinician burnout is a significant public health concern and has been associated with workforce-reducing factors. Using a random sample of nationally certified clinicians, we assessed clinician burnout and its potential association with increased sick days and intent to leave the profession. With more than 1800 respondents, clinicians reported high levels of burnout among 3 domains: personal (52%), work-related (49%), and patient-related (23%). Furthermore, presence of burnout indicated more than a 2-fold increase in reporting increased sick days and a 3-fold increase in intent to leave the profession.

## 1 Introduction

### 1.1 Background

Stress and strain on emergency medical services (EMS) clinicians, who often serve as the first point of care for individuals experiencing acute health concerns outside of a health care facility, have demonstrated workforce-reducing ramifications.^1^^,^^2^ Both globally and within the United States, EMS clinicians experience changes across their job tasks, including increasing operational demands, training needs, and personal protection concerns.^3^^,^^4^ Taken together, these developments have the potential of increasing strain on the EMS workforce. In response to the emergence of COVID-19, specifically, EMS clinicians experienced multiple challenges, including adapting to care requirements for patients with suspected or confirmed COVID-19, navigating COVID-19 vaccination recommendations, and managing inadequate staffing.^1^^,^^5^, ^6^, ^7^ Exacerbating these recent challenges were personal protective equipment shortages, COVID-19 vaccination resistance, workforce stability concerns, and attrition from EMS jobs.^8^, ^9^, ^10^

### 1.2 Importance

Many EMS clinicians report that ongoing job stress and the continuation of the COVID-19 pandemic continue to negatively affect their mental health, well-being, and job satisfaction. ^11^ Collectively, these ongoing and new experiences may result in increased risk of burnout, which has been linked to workforce-reducing factors such as absenteeism and turnover.^12^ Prior to the pandemic, burnout, seen as physical and emotional exhaustion, was observed within the EMS profession, with job demands (eg, time pressure, call volume) exceeding the available resources (eg, pay/benefits, culture, and training) needed to provide service.^12^^,^^13^ Without previous resolution, the pandemic intensified these concerns and revealed new potential sources of burnout.^1^ In addition, workflow changes, such as reduced interactions between colleagues (eg, as a result of minimizing personal interactions) reported by volunteer firefighters and emergency medical technicians (EMTs), have changed perspectives about workplace camaraderie.^14^ Considering these persistent and newly emerging pandemic-related concerns, continuing to assess and discuss burnout among EMS clinicians is critical to inform our understanding about how to protect their well-being and maintain the prehospital care infrastructure that relies on a strong and stable EMS workforce.

### 1.3 Goals of This Investigation

Although the stressors faced by EMS clinicians may contribute to higher turnover and burnout, there is limited evidence discussing recent burnout levels within the nationally certified EMS workforce. We sought to measure and contextualize burnout prevalence among EMS clinicians in a nationally representative sample and quantify associations with workforce-reducing factors.

## 2 Methods

### 2.1 Study Design, Population, and Setting

We conducted a cross-sectional evaluation of a random sample of nationally certified EMTs and paramedics aged 18 to 85 years. This evaluation asked participants about burnout and workforce-reducing factors. We collected data on our sample from the National Registry of Emergency Medical Technicians (National Registry), which provides initial and continuing EMS certification for the majority of states and territories in the United States and maintains the largest dataset of EMS clinicians with contact information for more than 500,000 EMS clinicians.^12^^,^^15^ As this evaluation aimed to assess the prevalence of burnout and identify characteristics associated with EMS workforce-reducing factors, only participants responding to burnout and workforce-reducing factor questions were included for analysis. The institutional review board of the American Institutes for Research approved this study and granted a waiver of consent.

### 2.2 Selection of Participants

We selected participants from the National Registry database, focusing on those certified prior to January 2020 to ensure equal exposure to the potential confounder of COVID-19. Based on our sample size calculation, which considered approximately 285,000 EMTs and 110,000 paramedics, we determined that 782 EMTs and 778 paramedic responses were needed to achieve estimates with 95% confidence and a 5% margin of error (±2.5%), assuming a conservative 50% outcome (workforce-reducing factors and absenteeism) prevalence. Anticipating that low response rates are common in electronic surveys of this population, we inflated our sample size, assuming an 8% response rate, and randomly selected 19,497 EMS clinicians for participation.^6^^,^^16^^,^^17^

### 2.3 Interventions: Survey and Data Collection

The survey tool was cognitively reviewed by practicing EMS clinicians for readability and consistency in item interpretation.^12^ The tool was distributed in April 2022 via the Alchemer survey platform (Alchemer LLC)^18^ and was titled “EMS Work-Life Survey” to mitigate response bias. Following a modified Dillman tailored design method, email reminders were sent 1 and 2 weeks after the initial survey invitation.^19^

An abbreviated nonresponder survey was also administered to all potential respondents who did not respond to survey invitations, with the intent to assess nonresponse bias. The abbreviated questionnaire asked EMS clinicians questions concerning the work-related Copenhagen Burnout Inventory (CBI) dimension and whether they had an intention to leave EMS in 12 months. This nonresponder survey was administered similarly to the main “EMS Work-Life Survey” following a single email.

### 2.4 Measurements

Demographic data were collected on the following variables as defined by the National Registry Database: age, sex (male or female), education level (high school or less, some college, associate’s degree, or bachelor’s degree or more), and race/ethnicity (White non-Hispanic or other). Because of the small proportion of EMS clinicians not identifying as White, non-Hispanic,^20^ race and ethnicity were dichotomized to White, non-Hispanic or other. The category of “other” was investigator-generated and included any person who self-identified as Black or African American, Asian, Hispanic or Latino, Asian or Native Hawaiian, or Pacific Islander. Employment characteristics included current certification level (EMT or paramedic), years of EMS experience, agency type (fire-based, private, hospital, government, military, or tribal), community size, volunteer EMS as their main job, weekly EMS call volume (<5, 5-9, 10-19, or ≥20 calls), and patient care delivery in the past 30 days (yes or no).

We measured burnout using the 19-item CBI, which has been adapted and employed in the EMS setting.^12^^,^^21^ The CBI assesses burnout, defined as the degree of physical and psychological fatigue and exhaustion, in 3 dimensions, including personal (related to that experienced by the person), work-related (perceived by the person as related to their work), and patient-related (perceived by the person as related to their work with patients). CBI is scored using a 5-point (always, often, sometimes, seldom, and never/or almost never) per question scale, with a composite score (eg, always = 100, sometimes = 50, and never = 0) calculated for each burnout dimension. For each dimension, an average score of ≥50 was considered positive for burnout.^12^ Patient-related burnout was calculated only for those individuals who reported providing patient care in the past 30 days.

### 2.5 Outcomes

Our outcomes of interest were sickness absence and anticipated profession turnover. Sickness absence was evaluated using 2 self-reported items: (1) the number of missed workdays due to personal illness over 12 months and (2) the number of missed workdays due to COVID-19 over 12 months. Using Bureau of Labor Statistics data on days of paid sick leave provided per year, we created the category of high number of annual workday absences being ≥10 days from personal illness.^22^ Similarly, we evaluated sickness absences from COVID-19 in 12 months using the a priori category of ≥5 due to the interim guidance from the Centers for Disease Control and Prevention (CDC) for returning to work for health care workers.^23^ We also evaluated anticipated turnover in 12 months using 2 items. The first item asked participants to indicate how likely they were to leave their main EMS job within the next 12 months (definitely will leave, probably will leave, probably will not leave, or definitely will not leave). The second item asked how likely they were to leave the EMS profession within the next 12 months. Responses to both of these items were dichotomized to those who indicated plans to definitely or probably leave vs those who did not.

### 2.6 Data Analyses

Survey results from respondents were merged with demographic characteristics from the National Registry dataset with removal of any identifying data. Descriptive statistics were calculated for demographics and EMS workforce characteristics. Proportions of EMS clinicians experiencing burnout were calculated for each dimension of the CBI. Missing data were handled via complete-case selection, with the highest areas being agency type (19%) and volunteer main EMS job (26%).

Two key research questions were evaluated: (1) the prevalence of burnout among EMS clinicians, and (2) the association between burnout and workforce-reducing factors. For the first, we calculated descriptive statistics, presented as frequency (n) and proportions (%) as appropriate. For the second, we used multivariable logistic regression modeling to assess association between burnout domains and workforce-reducing factors, including reporting more than 10 sick days over the past 12 months (yes/no) and intent to leave the EMS profession in the next 12 months (yes/no). Model selection was undertaken using a literature-informed process including variables previously identified as associated with workforce-reducing factors among similar population and sampling methodology evaluations.^12^^,^^24^ Odds ratios (ORs) and 95% CIs were calculated. Model fit was evaluated using the Hosmer-Lemeshow test.

## 3 Results

A total of 1838 of 19,497 (9%) respondents completed the survey, including 1220 paramedics and 618 EMTs. Respondent demographic and work-related characteristics are shown in Table 1. The abbreviated nonresponder survey had 953 respondents. Overall, EMS burnout prevalence was high for each specific dimension: personal (52%), work-related (49%), and patient-related (23%) (Table 2). Paramedics had a higher prevalence of burnout than EMTs in all domains. The abbreviated nonresponder survey showed higher (57% vs 49%) work-related burnout than that in the initial survey.

**Table 1:** Demographic and work-related characteristics of nationally certified emergency medical services clinicians, stratified by certification level for 2022 survey population.

Characteristic	Total (N = 1838), n (%)	EMT (n = 618), n (%)	Paramedic (n = 1220), n (%)
Provided patient care in last 30 d			
Yes	1508 (89)	481 (87)	1027 (90)
No	187 (11)	74 (13)	1113 (10)
Missing	143	63	80
Years of EMS experience			
<5	402 (22)	340 (55)	62 (5)
5-15	683 (37)	185 (30)	498 (41)
>15	753 (41)	93 (15)	660 (54)
Sex			
Female	497 (27)	228 (38)	269 (22)
Male	1324 (73)	380 (62)	944 (78)
Missing	17	10	7
Race/ethnicity			
White, non-Hispanic	1533 (87)	469 (81)	1064 (90)
Other	231 (13)	112 (19)	119 (10)
Missing	74	37	37
Agency type			
Fire	508 (34)	126 (34)	382 (33)
Private	374 (25)	81 (22)	293 (26)
Governmental nonfire	269 (18)	54 (15)	215 (19)
Hospital	213 (14)	43 (12)	170 (15)
Other	145 (10)	64 (17)	81 (7)
Missing	329	250	79
Shift length			
<12 h	211 (12)	78 (14)	133 (12)
12 h	530 (31)	197 (36)	333 (29)
≥24 h	818 (48)	198 (36)	620 (55)
Other	131 (8)	80 (14)	51 (4)
Missing	148	65	83
Weekly call volume			
<5 calls	400 (22)	175 (28)	225 (18)
5-9 calls	266 (14)	84 (14)	182 (15)
10-19 calls	427 (23)	127 (21)	300 (25)
20-29 calls	295 (16)	78 (13)	217 (18)
≥30 calls	450 (24)	154 (25)	296 (24)
Volunteer (main EMS job)			
Yes	5 (75)	42 (15)	33 (3)
No	1306 (95)	230 (85)	1076 (97)
Missing	457	346	111
Education			
High school or less	200 (11)	73 (12)	127 (10)
Some college	553 (30)	167 (27)	386 (32)
Associates degree	355 (19)	59 (10)	296 (24)
Bachelor’s degree or higher	730 (40)	319 (52)	411 (34)
Sickness absence			
<10 d	1522 (83)	491 (79)	1031 (85)
≥10 d	316 (17)	127 (21)	189 (15)
Sickness absence due to COVID-19			
<5 d	480 (26)	150 (24)	330 (27)
≥5 d	1358 (74)	468 (76)	890 (73)
Intent to leave EMS job within 12 mo			
Unlikely	1194 (71)	364 (66)	830 (73)
Likely	496 (29)	189 (34)	307 (27)
Missing	148	65	83
Intent to leave EMS profession within 12 mo			
Unlikely	1407 (83)	447 (81)	960 (85)
Likely	281 (17)	106 (19)	175 (15)
Missing	150	65	85

EMS, emergency medical services; EMT, emergency medical technician.

**Table 2:** Prevalence of each burnout domain among nationally certified emergency medical technicians and paramedics initially sampled and work-related burnout among those responding to the nonresponder survey.

Burnout division	Total (N = 1838) % (95% CI)	Paramedic (n = 1220) % (95% CI)	EMT (n = 618) % (95% CI)	Nonresponder Total (n = 953) % (95% CI)
Personal	52 (50-55)	54 (51-56)	49 (45-53)	n/a
Work-related	50 (48-52)	52 (49-55)	46 (42-50)	57 (55-59)
Patient-related	23 (21-25)	27 (24-30)	15 (12-18)	n/a

EMT, emergency medical technician; n/a, not applicable.

Multivariable logistic regression revealed that sample respondents who reported personal (OR, 2.66; 95% CI, 1.70-4.15), work-related (OR, 1.99; 95% CI, 1.31-3.01), or patient-related burnout (OR, 1.85; 95% CI, 1.20-2.86) had greater odds of reporting having missed 10 or more days of work due to sickness in the past 12 months (Table 3). A more than 3-fold increase in odds of intent to leave the EMS profession was observed for those with personal (OR, 3.06; 95% CI, 2.16-4.15), work-related (OR, 3.34; 95% CI, 2.35-4.74), or patient-related burnout (OR, 3.42; 95% CI, 2.39-4.90). Furthermore, nonresponders showed higher (20% vs 17%) intent to leave the profession than those who initially responded.

**Table 3:** Association between presence of burnout domains and odds of reporting 10 or more sickness absences in the last 12 months or intent to leave the EMS profession in the next 12 months using multivariable logistic regression models.

Characteristic	Odds ratio (95% CI)	*P* value
≥10 sickness absence days in last 12 mo^a^^,^^b^		
Personal burnout	2.66 (1.70-4.15)	<.001
Work-related burnout	1.99 (1.31-3.01)	.001
Patient-related burnout	1.85 (1.20-2.86)	.006
Likely to leave EMS profession within next 12 mo^a^^,^^b^		
Personal burnout	3.06 (2.16-4.33)	<.001
Work-related burnout	3.34 (2.35-4.74)	<.001
Patient-related burnout	3.42 (2.39-4.90)	<.001

EMS, emergency medical services.

a Adjusted for sex, race and ethnicity, agency type, certification level, weekly call volume, EMS experience, and educational level.

b Hosmer-Lemeshow goodness-of-fit *P* values of >.05.

## 4 Limitations

This study was limited to EMS clinicians who were nationally certified. Nearly all states require national certification as part of their requirements for initial certification; however, as of 2021, only 9 states require continued maintenance of national certification. Thus, this sample is weighted toward nationally certified clinicians who may differ from clinicians who are not certified, potentially influencing external generalizability. Furthermore, our sample may suffer from selection bias of unknown directionality, as evidenced by a low response rate and potential sample-to-population differences influenced by sampling bias, seen here with a higher education level among this sample compared with previous National Registry demography data.^2^ Our brief nonresponder survey, used to briefly quantify nonresponding individuals, revealed differences in work-related burnout, possibly indicating uncaptured bias. Additionally, the time frame of data collection paralleled the progression of the COVID-19 pandemic, and although some measure of burnout prevalence could be associated with this, the strength of that association is unknown. It should be noted that, using the Johns Hopkins dashboard of confirmed cases, the timing of our sampling was not affected by any particular surge in COVID-19 cases. Lastly, this research design detects associations between burnout and workforce-reducing factors, not causal ties.

## 5 Discussion

Maintenance of a stable, competent workforce to care for US citizens who require prehospital care from EMS clinicians is critically important. In this evaluation, we found high prevalences of burnout among EMS clinicians. Specifically, we detailed differing domains of burnout (personal, work-related, and patient-related) among EMS clinicians and found associations between these domains and workforce-reducing outcomes of increased sickness days and intent to leave the EMS workforce.

Our findings are concerning for several reasons. First, our results show that burnout prevalence is alarmingly high in our sample. Compared with a previous sample taken from the National Registry population in 2015, burnout has nearly doubled in 2 of the 3 measured domains.^12^ In additional studies prior to the COVID-19 pandemic, EMS personnel and first responders reported high levels of burnout and posttraumatic stress disorder.^12^^,^^25^^,^^26^ Notably, in 2022, the Surgeon General published an advisory concerning health care worker burnout and increased rates of suicide among nurses and physicians.^27^ Our results show that a high prevalence of burnout exists among EMS clinicians, suggesting a continued need for attention to this workforce as well. Furthermore, although we found that all EMS clinicians studied experienced high rates of burnout, prevalence of burnout was more pronounced among paramedics.

The degree to which the 2020 onset and continuation of the COVID-19 pandemic have contributed to burnout and workforce-reducing factors at the time of this survey distribution is uncertain but is presumed to play a significant role. COVID-19–related burnout has been documented among various frontline health care workers, including smaller, localized samples of EMS personnel.^28^, ^29^, ^30^, ^31^ Of note, most of these prior studies have focused exclusively on the pandemic period, with fewer studies examining changes in separately defined time periods. However, in a study of physician burnout comparing prepandemic levels with pandemic levels, Shanafelt et al^25^ found a marked increase in burnout in 2021 compared with data from previous years (2011 and onward).

Results from this study further underscore the need to address burnout in the EMS community and to identify potential barriers that impede EMS clinicians from requesting assistance at all experience levels, which may help them better cope with stress. When individuals perceive their burnout experiences as unique to themselves, this can discourage them from speaking out about their experiences or even seeking any assistance.^32^ Communication campaigns that highlight the broad problem of burnout as well as the resources available to address it may be important in mitigating the issue of EMS clinician burnout.

National evaluations of EMS clinicians demonstrate overall that turnover is high. In our study, 15% of EMS clinicians reported an intent to leave the profession within the next 12 months, highlighting the workforce’s reliance on a steady influx of new trainees to offset the significant stressors embedded within the system.^7^ Combined with our findings about high burnout and workforce-reducing factors, as well as the association between burnout and likeliness of leaving the profession, such evidence suggests that we may be approaching an EMS shortage that could jeopardize prehospital, emergency, and public health systems. The United States Bureau of Labor Statistics estimates a 7% growth rate for EMT and paramedic jobs in the next 10 years on the basis of transfers and retirement.^33^ However, if more EMS clinicians leave the profession in part due to burnout, it is unknown if the usual pipeline for replacement through postsecondary education, certification, and licensure will maintain capacity for adequate public safety. This situation echoes a trend seen among nurses and physicians, where almost one-third to one-half of professional nurses and one-fifth of physicians indicated their intent to leave direct patient care.^34^^,^^35^ Of note, the National Conference of State Legislatures reports considering several actions to improve the recruitment and retention of EMS personnel, although the implementation of these actions has yet to see dividends.^36^ Leveraging local, state, and federal stakeholders for continued assessment and intervention will be critical to maintain a healthy and stable EMS workforce.

Nationally certified EMS clinician demonstrated high burnout in 2022, likely influenced by existing workplace strain and emerging stressors due to the COVID-19 pandemic in 2020. Combined with increased absenteeism and intent to leave the profession associated with these higher levels of burnout, our findings suggest that a renewed and deliberate focus on EMS clinician well-being is needed to ensure job satisfaction and workforce stability.

## Author Contributions

JRP, RPC, CBG, ASM, and ARP conceived and designed the study. JRP, RPC, CBG, and ARP collected the data. JRP, CBG, ARP analyzed and interpreted the data. JRP, ARP, SRM, LJR, GD, and ASM drafted the manuscript. All authors contributed substantially to the revision of the manuscript. JRP takes responsibility for the paper as a whole.

## Funding and Support

This study was supported by a grant (number CA260582) from the 10.13039/100000054National Cancer Institute (NCI), but the NCI was not involved in the data collection, analysis, or decision to present these findings.

## Conflict of Interest

All authors have affirmed they have no conflicts of interest to declare.

## References

[1] Melnyk H., Di Tosto G., Powell J., Panchal A.R., McAlearney A.S. (2023). Conflict in the EMS workforce: an analysis of an open-ended survey question reveals a complex assemblage of stress, burnout, and pandemic-related factors influencing well-being. Int J Environ Res Public Health.

[2] Rivard M.K., Cash R.E., Mercer C.B., Chrzan K., Panchal A.R. (2021). Demography of the national emergency medical services workforce: a description of those providing patient care in the prehospital setting. Prehosp Emerg Care.

[3] Al Amiry A., Maguire B.J. (2021). Emergency medical services (EMS) calls during COVID-19: early lessons learned for systems planning (a narrative review). Open Access Emerg Med.

[4] Cash R.E., Rivard M.K., Camargo C.A., Powell J.R., Panchal A.R. (2021). Emergency medical services personnel awareness and training about personal protective equipment during the COVID-19 pandemic. Prehosp Emerg Care.

[5] Ventura C.A.I., Denton E.E., David J.A. (2022). Emergency medical services prehospital response to the COVID-19 pandemic in the US: a brief literature review. Open Access Emerg Med.

[6] Gregory M.E., Powell J.R., MacEwan S.R. (2022). COVID-19 vaccinations in EMS professionals: prevalence and predictors. Prehosp Emerg Care.

[7] Kurth J.D., Powell J.R., Gage C.B. (2023). Evaluating changes in the emergency medical services workforce: a preliminary multistate study. J Am Coll Emerg Physicians Open.

[8] American Ambulance Association 4th annual study shows worsening EMS turnover. https://ambulance.org/2022/10/17/4th-annual-study-shows-worsening-ems-turnover/.

[9] Hubble M.W., Renkiewicz G.K., Hunter S., Kearns R.D. (2022). Predictors of COVID-19 vaccination among EMS personnel. West J Emerg Med.

[10] Ehrlich H., McKenney M., Elkbuli A. (2021). Defending the front lines during the COVID-19 pandemic: protecting our first responders and emergency medical service personnel. Am J Emerg Med.

[11] Brown G.D., Largey A., McMullan C., O'Shea G., Reilly N. (2023). Voices from the frontline: a review of EMS first responders’ experience of COVID-19 in Ireland. Int J Emerg Serv.

[12] Crowe R.P., Bower J.K., Cash R.E., Panchal A.R., Rodriguez S.A., Olivo-Marston S.E. (2018). Association of burnout with workforce-reducing factors among EMS professionals. Prehosp Emerg Care.

[13] Crowe R.P., Fernandez A.R., Pepe P.E. (2020). The association of job demands and resources with burnout among emergency medical services professionals. J Am Coll Emerg Physicians Open.

[14] Gaughan A.A., Rush L.J., MacEwan S.R., Panchal A.R., McAlearney A.S. (2022). Perspectives of volunteer firefighters during the COVID-19 pandemic: stumbling blocks and silver linings. Challenges (Basel).

[15] National Registry of Emergency Medical Technicians National registry data, dashboard, & maps. https://nremt.org/maps.

[16] Cash R.E., White-Mills K., Crowe R.P., Rivard M.K., Panchal A.R. (2019). Workplace incivility among nationally certified EMS professionals and associations with workforce-reducing factors and organizational culture. Prehosp Emerg Care.

[17] Panchal A.R., Rivard M.K., Cash R.E. (2022). Methods and implementation of the 2019 EMS practice analysis. Prehosp Emerg Care.

[18] Alchemer Online survey platform. https://www.alchemer.com/.

[19] Dillman D.A., Smyth J.D., Christian L.M. (2014).

[20] Crowe R.P., Krebs W., Cash R.E., Rivard M.K., Lincoln E.W., Panchal A.R. (2020). Females and minority racial/ethnic groups remain underrepresented in emergency medical services: a ten-year assessment, 2008-2017. Prehosp Emerg Care.

[21] Kristensen T.S., Borritz M., Villadsen E., Christensen K.B. (2005). The Copenhagen Burnout Inventory: a new tool for the assessment of burnout. Work Stress.

[22] US Bureau of Labor Statistics Number of paid sick leave days in 2015 varies by length of service and establishment size. https://www.bls.gov/opub/ted/2016/number-of-paid-sick-leave-days-in-2015-varies-by-length-of-service-and-establishment-size.htm.

[23] Centers for Disease Control and Prevention Interim guidance for managing healthcare personnel with SARS-CoV-2 infection or exposure to SARS-CoV-2. https://www.cdc.gov/covid/hcp/infection-control/guidance-risk-assesment-hcp.html?CDC_AAref_Val=https://www.cdc.gov/coronavirus/2019-ncov/hcp/guidance-risk-assesment-hcp.html.

[24] Hosmer D.W., Lemeshow S., Sturdivant R.X. (2013).

[25] Shanafelt T.D., West C.P., Dyrbye L.N. (2022). Changes in burnout and satisfaction with work-life integration in physicians during the first 2 years of the COVID-19 pandemic. Mayo Clin Proc.

[26] Klimley K.E., Van Hasselt V.B., Stripling A.M. (2018). Posttraumatic stress disorder in police, firefighters, and emergency dispatchers. Aggression Violent Behav.

[27] Office of the Surgeon General Addressing health worker burnout. https://www.hhs.gov/sites/default/files/health-worker-wellbeing-advisory.pdf.

[28] National Academy of Medicine Clinician burnout crisis in the era of COVID-19: insights from the frontlines of care. https://nam.edu/initiatives/clinician-resilience-and-well-being/clinician-burnout-crisis-in-the-era-of-covid-19/.

[29] Prasad K., McLoughlin C., Stillman M. (2021). Prevalence and correlates of stress and burnout among U.S. healthcare workers during the COVID-19 pandemic: a national cross-sectional survey study. EClinicalMedicine.

[30] Petrino R., Riesgo L.G.C., Yilmaz B. (2022). Burnout in emergency medicine professionals after 2 years of the COVID-19 pandemic: a threat to the healthcare system?. Eur J Emerg Med.

[31] Blanchard J., Li Y., Bentley S.K. (2022). The perceived work environment and well-being: a survey of emergency health care workers during the COVID-19 pandemic. Acad Emerg Med.

[32] Sargent R.H., Newman L.S. (2021). Pluralistic ignorance research in psychology: a scoping review of topic and method variation and directions for future research. Rev Gen Psychol.

[33] Bureau of Labor Statistics, US Department of Labor Occupational *Outlook Handbook*, EMTs and Paramedics. https://www.bls.gov/ooh/healthcare/emts-and-paramedics.htm.

[34] Berlin G., Lapointe M., Murphy M. Surveyed nurses consider leaving direct patient care at elevated rates. https://www.mckinsey.com/industries/healthcare/our-insights/surveyed-nurses-consider-leaving-direct-patient-care-at-elevated-rates.

[35] LeClaire M., Poplau S., Linzer M., Brown R., Sinsky C. (2022). Compromised integrity, burnout, and intent to leave the job in critical care nurses and physicians. Crit Care Explor.

[36] George K., Sweeney S. State actions to address EMS workforce shortage. https://www.ncsl.org/health/state-actions-to-address-ems-workforce-shortages.

